# Social constructivist analysis of cultural concepts of distress and lycanthropy in Nagaland India

**DOI:** 10.1007/s44192-025-00295-2

**Published:** 2025-11-13

**Authors:** Saranya TS, Kiniholi Yepthomi

**Affiliations:** 1 Amity Institute of Behavioural Health and Allied Science, Amity University Bengaluru, Bengaluru, India; 2 Research Scholar, School of Liberal Studies, CMR University, Bengaluru, India

**Keywords:** Modern influences, Traditional healing practices, Mythology and mental illness, Supernatural beliefs in psychology, Tribal identity and distress, Spirit possession and mental health, Shapeshifting and cultural narratives

## Abstract

This systematic review examines how cultural constructs of distress and the phenomenon of lycanthropy are understood within the context of Nagaland, emphasizing a social constructivist framework. A comprehensive literature search was conducted across Google Scholar, PubMed, and PsycINFO, ultimately shortlisting 24 methodologically sound studies relevant to cultural interpretations of mental health. Data extraction focused on themes such as spiritual beliefs, indigenous healing practices, the cultural framing of lycanthropy, and the role of modernization in shaping local perceptions of distress. Findings indicate that Nagaland’s cultural narratives and communal belief systems strongly influence how distress is experienced, conceptualized, and addressed. Lycanthropy, often perceived as a delusional disorder in Western psychiatry, is regarded by many communities in Nagaland as a spiritual affliction or culturally rooted manifestation of distress. Moreover, traditional healers and ritual practices continue to play a critical role in managing mental health concerns, even as modern psychiatric approaches gain visibility. By highlighting the intersection of cultural beliefs, supernatural interpretations, and communal coping strategies, this review emphasizes the importance of integrating indigenous perspectives with contemporary mental health care. Such culturally sensitive, hybrid interventions can bridge long standing traditions with modern practices, ultimately fostering more holistic and effective approaches to mental well-being in Nagaland.

## Introduction

Cultural Constructs of Distress and Lycanthropy Cultural constructs of distress refer to culturally specific ways of understanding and interpreting psychological suffering, often grounded in localized belief systems, traditions, and societal norms [15]. These constructs shape how individuals and communities perceive mental health issues, seek help, and respond to treatment. Social constructivism, a theoretical framework developed by Berger and Luckmann [4], posits that realities are constructed through social interactions and shared meanings [4]. This perspective emphasizes the notion that concepts of normality and abnormality are not universal but context-dependent [8].

In the context of Nagaland, a region with rich cultural diversity and indigenous belief systems, interpretations of mental health and distress are deeply intertwined with traditional folklore, animistic beliefs, and socio-cultural narratives. Among these, the concept of lycanthropy—commonly understood as the delusion of transforming into a wolf or other animals—holds unique cultural significance. Unlike its portrayal in Western psychiatric literature as a rare delusional disorder [6], lycanthropy in Nagaland may be perceived through a cultural lens as a manifestation of spiritual disturbances, ancestral discontent, or supernatural influence [20]. These interpretations are often influenced by narratives passed down through generations, local healers’ perspectives, and communal belief systems.

This systematic review seeks to explore the cultural constructs of distress and lycanthropy in Nagaland, analyzing how these constructs influence individuals’ lived experiences, coping mechanisms, and help-seeking behaviors (Kirmayer & Bhugra, 2021). By adopting a social constructivist perspective, the review aims to deconstruct the ways in which societal norms, traditional practices, and modern influences intersect to shape understandings of mental health and abnormal behavior [4]. The analysis will also examine the role of indigenous healing practices, the impact of modernization, and the implications for culturally sensitive mental health interventions in the region [20].

Drawing from Kaiser & Jo Weaver [14], lycanthropy in Nagaland can be conceptualized as an idiom of distress or culture-bound syndrome, as opposed to being just a delusional disorder. This places it within wider debates on cultural concepts of distress. Ultimately, this research aspires to bridge the gap between culturally rooted understandings of distress and contemporary psychological frameworks, offering insights for mental health practitioners, policymakers, and researchers working in culturally diverse settings [8, 15].

### Search and inclusion strategy

To systematically review the Nagaland’s cultural constructs of lycanthropy and distress, a combined inclusion and search strategy was adopted. A thorough literature search across PsycINFO, Google Scholar, and PubMed was undertaken, which collectively cover comprehensive peer-reviewed articles on psychology, anthropology, and cultural studies.

The search strategy employed combinations of keywords like “cultural constructs of distress,” “lycanthropy and culture,” “mental health beliefs in Nagaland,” “indigenous healing practices,” and “social constructivist analysis of mental health.” Pertinent ethnographic studies, case reports, and qualitative research on supernatural beliefs and mental health in Northeast India were also utilized.

**Studies were included if they**:

Directed towards cultural constructs of distress, supernatural beliefs, or indigenous views of mental health, with a focus on Nagaland or closely linked cultural settings.

Used qualitative, quantitative, or mixed-method studies that provided empirical or ethnographic findings on the sociocultural aspects of lycanthropy or distress.Were peer-reviewed materials (journal articles, books, or conference proceedings) to ensure academic integrity and credibility.

For methodological quality assurance, inclusion was limited to studies considered methodologically robust. As per the advice of Kohrt et al. [17, 19], this decision was based on sampling transparency, triangulation of data sources, clarity of the cultural setting, and peer review evidence.

Studies were to be excluded if they (a) considered only clinical psychiatric understandings of lycanthropy without involving cultural or social constructivist approaches, (b) were not peer reviewed (e.g., blogs, unpublished manuscripts), or (c) were published prior to 2014 unless of historical significance to the subject. This dual approach guaranteed that the ultimate review contained a methodologically sound, interdisciplinary, and culturally informed set of literature, capturing insights from psychology, anthropology, and sociology.

### Exclusion criteria

To maintain the focus and reliability of the review, studies were excluded based on the following criteria:

- Research that solely examined clinical psychiatric interpretations of lycanthropy without considering cultural or social constructivist perspectives.
- Articles not subject to peer review, such as blog posts, opinion pieces, and unpublished manuscripts.
- Studies published before 2014, unless they were of significant relevance to understanding historical perspectives on lycanthropy and cultural distress.
- Research that did not directly relate to Nagaland or similar indigenous communities with parallel beliefs and practices.

By applying these exclusion criteria, the review ensures a focused, high-quality synthesis of literature that highlights the intersection of cultural beliefs, supernatural interpretations, and mental health in Nagaland.

### Data extraction and analysis

Following the selection of studies, data were extracted and organized into thematic categories that aligned with the research objectives of understanding cultural constructs of distress and lycanthropy in Nagaland. This systematic categorization facilitated a comprehensive evaluation of the literature. The thematic categories included:

- **Cultural Constructs of Distress**: This category examined various cultural interpretations of mental health distress within the context of Nagaland. It focused on how spiritual disturbances, ancestral discontent, or supernatural beliefs (e.g., spirit possession) were understood as forms of psychological suffering. Studies in this category analyzed how these cultural constructs influence individuals’ perceptions of distress and their help-seeking behaviors.
- **Lycanthropy and Its Cultural Significance**: Studies in this category explored the concept of lycanthropy, particularly within the cultural context of Nagaland, where the belief in transformation into wolves or other animals is linked to spiritual beliefs and social conditions. This theme also focused on how the experience of lycanthropy is viewed not as a psychiatric disorder but rather as a manifestation of cultural, spiritual, or social distress.
- **Traditional Healing Practices and Modern Psychological Interventions**: This theme addressed the role of indigenous healing practices in managing distress, including the use of local healers and spiritual rituals. Additionally, the influence of modern psychiatric approaches and their integration with traditional belief systems was explored, focusing on how these dynamics affect the treatment and perception of mental health issues, including lycanthropy.

The systematic organization of data into these categories allowed for an in-depth understanding of how cultural constructs shape the lived experiences of distress in Nagaland and their implications for mental health interventions.

### Results of the search process

The systematic search process yielded a large number of initial records:

- **Google Scholar**: 19,500 records.
- **PubMed**: 92 records.
- **PsycINFO**: 45 records.

These records were screened based on their relevance to the research questions, their peer-reviewed status, and their adherence to the inclusion criteria specified earlier. After the initial screening, the following records were shortlisted for further evaluation:

- **Google Scholar**: 120 records.
- **PubMed**: 18 records.
- **PsycINFO**: 10 records.

Subsequent evaluation led to the exclusion of:

- **Google Scholar**: 14,380 records, primarily due to lack of relevance, absence of peer review, or non-compliance with the specified timeframe (2014–2024).
- **PubMed**: 62 records, based on similar exclusion criteria.
- **PsycINFO**: 35 records, due to relevance and methodological concerns.

**Fig. 1:**
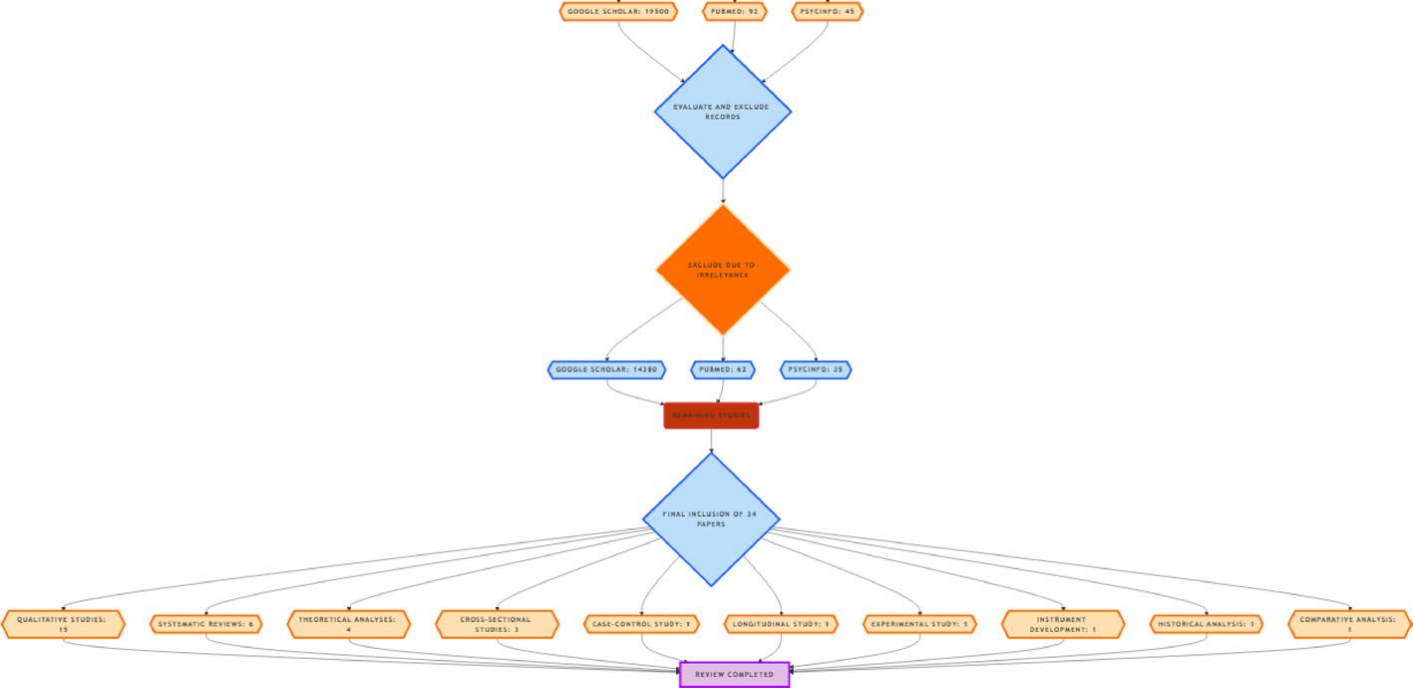
Flow chart of screening process

After the screening and evaluation process, a final count of **34 studies** was selected for detailed analysis. These studies were deemed highly relevant, methodologically sound, and aligned with the research objectives of exploring the cultural constructs of distress and lycanthropy in Nagaland. The selected studies provided valuable insights into the intersection of culture, mental health, and traditional practices in the region (Fig. 1).

### Disciplinary distribution of literature

The 34 studies represented here cut across psychology, anthropology, and sociology, mirroring the intrinsically interdisciplinary character of cultural psychiatry studies. Of the 34 studies, 15 were grounded in psychology, addressing cultural psychiatry, idioms of distress, and clinical case studies of lycanthropy; 12 were anthropological, highlighting ethnographic descriptions, indigenous healing, and community cosmologies; and 7 were sociological, examining the impact of modernization, stigma, and collective coping mechanisms on mental illness in Nagaland.

This disciplinary distribution emphasizes that although psychology offers therapeutic and diagnostic insights, anthropology situates the debate in local cosmologies and healing practices, and sociology adds clues to the general social structures shaping mental well-being. Combined, these insights emphasize the necessity of integrative models that explain both individual psychopathology and collective cultural meaning-making.

The following sections present the results thematically, building upon the disciplinary distribution outlined above:

## Results and discussion

This section presents the findings of the systematic review on the cultural constructs of distress and lycanthropy in Nagaland, focusing on the social constructivist perspective. The findings are organized into thematic categories that emerged from the literature, followed by a discussion of their implications in understanding mental health in this culturally diverse region.

### Cultural constructs of distress in Nagaland

The studies included in this review highlighted several key cultural constructs that shape the perception of distress in Nagaland. These constructs are deeply embedded in the indigenous belief systems and folklore, where mental health issues are often understood through the lens of spiritual disturbances, ancestral discontent, and supernatural forces (Table 1).

1. **Spiritual and Supernatural Beliefs** .
2. A significant finding was the role of spiritual beliefs in interpreting distress. Many studies suggested that psychological suffering in Nagaland is often attributed to supernatural forces, including spirits or ancestral anger [20]. Such beliefs influence how individuals and families perceive mental health issues and seek help from local healers or through ritualistic practices. These findings are consistent with Kirmayer and Bhugra’s (2021) argument that cultural interpretations of mental health often reflect broader societal norms and religious values.
3. **Traditional Healing Practices** .
4. The role of traditional healers, including shamans and local spiritual leaders, was another key finding. Many Naga communities rely on these figures to address mental health issues, with treatment often involving rituals, prayers, and offerings to appease spirits or ancestors. While these practices provide cultural continuity and social cohesion, their integration with modern psychological frameworks presents a challenge for mental health practitioners in the region.

**Table 1:** Key studies on cultural constructs of distress in Nagaland and broader cultural psychiatry frameworks

Author & year	Title	Key findings	Critical notes
Longkumar [20]	Traditional Healing Practices and Perspectives of Mental Health in Nagaland	Highlights the role of ancestral beliefs and spirits in explaining distress, with implications for local healing practices.	Rich ethnographic insight into healing rituals, but findings are context-specific to select tribes and lack broader generalizability.
Kleinman [16],	Rethinking Cultural Psychiatry: Contextual Perspectives	Kleinman focuses on the importance of personal experience in understanding mental illness. He asserts that the experience of illness is not just biological but also deeply personal and shaped by cultural frameworks. The meaning of illness varies, and individuals interpret and experience their mental health struggles in ways that are informed by their cultural surroundings.	Seminal theoretical work, highly cited, but dated. Does not engage with Northeast India directly applied here for conceptual grounding.
Guessoum, Benoit, Minassian, Mallet, & Moro [10],	Clinical Lycanthropy, Neurobiology, Culture: A Systematic Review	The review also highlights the influence of cultural beliefs and folklore on the manifestation of clinical lycanthropy. It discusses the association of the condition with psychiatric disorders like schizophrenia and dissociative identity disorder, and presents case studies showing cultural variations. Treatment approaches focus on pharmacological and psychological interventions to address underlying conditions.	Provides cross-cultural perspective, but relies heavily on clinical psychiatry. Limited engagement with indigenous interpretations like those in Nagaland.

### Lycanthropy as a cultural phenomenon

One of the most intriguing findings of this review was the interpretation of lycanthropy in Nagaland. While lycanthropy is typically understood as a psychiatric disorder in Western contexts, characterized by the delusional belief that one can transform into an animal [6], in Nagaland, it is often seen as a manifestation of spiritual distress.These signs spiritual disturbances, ancestral discontent, affliction, and supernatural influence are not optimized as separate categories but as interrelated expressions of lycanthropy within Naga cultural explanations. The literature is such that they co-occur as interlocking interpretive perspectives rather than separable causes.

**Cultural Interpretation of Lycanthropy** Studies revealed that lycanthropy in Nagaland is frequently linked to spiritual disturbances, such as the anger of ancestors or punishment for past wrongdoings. These interpretations are deeply rooted in Naga folklore, where the belief in shape-shifting into animals is common in various indigenous tribes. The review suggests that this cultural lens provides a unique understanding of what might otherwise be seen as a mental health disorder in the Western diagnostic model.

**Role of Social Norms in Understanding Lycanthropy** The social context within which lycanthropy is understood plays a critical role in its manifestation. In some cases, lycanthropy is seen as a form of punishment for individuals who have transgressed social or moral norms, leading to a supernatural “curse” [13]. This view emphasizes the influence of communal values and the collective belief in ancestral power, highlighting how social norms are internalized and shape individual behavior.

**Persistence Amid Modernization** Sutter’s [23] research on ‘tigermen’ (‘tekhumiavi’) demonstrates how these beliefs endure despite significant Christianization and modernization in Naga communities. Her study traces the development of the Naga concept of human-tiger transformation from traditional practices to contemporary expressions, revealing the remarkable adaptability of these beliefs in negotiating between tradition and modernity.

**Identity Preservation Through Supernatural Beliefs** Aye’s [3] examination of Sümi lycanthropy illustrates how these supernatural beliefs contribute to sustaining tribal identity in the face of globalization pressures. The study suggests that shape-shifting narratives serve as cultural repositories that help preserve indigenous cosmologies while simultaneously allowing communities to make sense of social changes.

**Structural Functions of Lycanthropic Beliefs** Ovesen’s [21] analysis reveals how lycanthropy beliefs in the Naga Hills reflect and reinforce social hierarchies and categorizations. His work demonstrates that the ambiguous boundary between human and animal forms in Naga cosmology mirrors complex social relationships and power structures within these communities, providing insights into how supernatural beliefs articulate with social organization (Table 2).

**Table 2:** Key studies on lycanthropy in Nagaland

Author & year	Title	Key findings	Critical notes
Hutton [13]	The Angami Nagas and The Sema Nagas. Macmillan.	John Henry Hutton was a renowned British administrator and anthropologist who conducted extensive research on Naga tribes during his tenure in the Naga Hills. His monographs, *The Angami Nagas* and *The Sema Nagas*, published in 1921, are seminal works in Naga ethnography. Hutton’s contributions to Naga studies are well-documented, and his collections are housed in institutions like the Pitt Rivers Museum.	A foundational text for Naga studies, but dated and framed within colonial anthropology. Requires careful contextualization when used today.
Heneise [12]	Agency and Knowledge in Northeast India: The Life and Landscapes of Dreams	Michael Heneise’s work explores how dreams among the Angami Naga are significant avenues for negotiating uncertainty and dealing with the future. Through dreams and dreaming, individuals may glean knowledge from signs, gain insights from ancestors, and potentially obtain divine blessings. This shows the importance of cultural narratives in understanding mental health experiences within the Naga community.	Offers a nuanced cultural lens, though not focused exclusively on lycanthropy; relevance lies in showing broader spiritual frameworks of distress.
Aye [3]	Human-Animal Transitions: A Case Study of Sümi Lycanthropy	This study examines the belief in lycanthropy among the Sümi Naga, exploring its socio-religious implications and its role in sustaining tribal identity amidst globalization.	Strong recent ethnographic contribution, but limited to a single Naga subgroup. Needs comparative extension across tribes.
Sutter [23]	Collective imagination or intimate knowledge of other worlds?	This study explores the enduring belief in ‘tigermen’ among the Naga communities of Nagaland, India. Despite significant Christianization, this belief persists, blending folklore with personal experiences. Sutter examines the cultural and social dimensions of this belief, tracing the development of the Naga ‘tekhumiavi’ (person in the shape of a tiger) from traditional practices to modern myth. She highlights the interplay between indigenous beliefs and external influences, providing insights into how such beliefs shape social structures, identity, and the negotiation between tradition and modernity within Naga communities.	Richly contextualized; highlights adaptability of beliefs. However, largely descriptive with limited engagement in mental health implications.
Hutton, (2023)	Black Magic, Witchcraft and Occultism	J.H. Hutton contributes a chapter titled “Some Astronomical Beliefs in the North-East and other Parts of India.” In this chapter, Hutton explores traditional astronomical beliefs prevalent among communities in Northeast India and other regions, shedding light on how these beliefs intertwine with local cultural and occult practices	Provides historical-cultural context, but is more descriptive of rituals than directly tied to lycanthropy.
Ovesen [21], .	Man or Beast? Lycanthropy in the Naga Hills	The journal explores the cultural significance of lycanthropy among the Naga communities. Ovesen’s research is cited in various academic works, understanding its scholarly impact.	Useful sociological lens, but based on limited case material; generalizations should be made cautiously.
Bettini [5],	The Rage of the Wolf: Metamorphosis and Identity in Medieval Werewolf Tales	This study analyzes medieval werewolf narratives, emphasizing how transformations are depicted as consequences of moral and spiritual transgressions.	Provides valuable cross-cultural comparison, but not specific to Nagaland; applied here to broaden conceptual parallels.

### Coping mechanisms and Help-Seeking behaviors

The review also examined how Naga individuals cope with distress, particularly in relation to lycanthropy. The findings suggest that coping mechanisms are largely influenced by cultural beliefs, with individuals often seeking help from traditional healers rather than mental health professionals (Table 3).

1. **Rituals and Community Support**.
2. Rituals performed by spiritual healers were found to be a central coping mechanism for individuals experiencing distress or lycanthropy. These rituals are seen not only as healing practices but also as communal events that reinforce cultural identity and provide a sense of belonging. Furthermore, community support plays a vital role in coping, with extended families and local networks often involved in the care and support of individuals facing mental health challenges.
3. **Challenges in Accessing Modern Mental Health Services**.
4. A recurring theme in the studies was the difficulty of accessing modern mental health services in Nagaland. There is a lack of mental health infrastructure in rural areas, and the stigma associated with mental illness often prevents individuals from seeking help outside their communities (Kirmayer & Bhugra, 2021). This lack of integration between traditional and modern healthcare systems creates a gap in providing effective mental health care.

**Table 3:** Key studies on coping mechanisms and Help-Seeking behaviors

Author & year	Title	Key findings	Critical notes
Lolly Yeptho & Harikrishnan	Socio-Cultural Factors and Mental Illness in North-Eastern Region of India: A Review	The review highlights how traditional healing practices, social stigma, and limited mental health care access affect mental health in North-East India. It emphasizes the need for integrating evidence-based treatments with culturally sensitive approaches, recognizing the role of social workers in bridging the gap between traditional and modern mental health interventions.	Provides a broad regional perspective; however, its scope is a literature review rather than primary data.
Watienla, & Jamir [24]	Indigenous Health Practices of the Naga People: Continuity and Change	This paper reviews the holistic health practices of the Naga people, emphasizing a balanced state of physical, mental, spiritual, and communal well-being. The authors document traditional healing methods—including natural remedies from plants, animals, and minerals, alongside spiritual healing rituals—that historically sustained community health. They also discuss the contemporary coexistence of traditional and modern medical practices, raising questions about the relevance and preservation of indigenous healing methods amid ongoing modernization and globalization.	Valuable ethnographic evidence on indigenous practices. Limitation: descriptive in nature.
Kirmayer, & Bhugra [15]	Rethinking Cultural Psychiatry: Contextual Perspectives	Highlights the challenges rural communities face in accessing mental health services and the role of traditional healers.	Offers a global theoretical framework applied to Nagaland. While conceptually strong, lacks direct empirical data from the region.
Blom, & Hoffer [6]	Lycanthropy Revisited: Delusion or Cultural Belief?	Explores how cultural beliefs influence help-seeking behavior for mental health issues like lycanthropy.	Useful for linking lycanthropy to psychiatric debates, but empirical cases are drawn mainly from outside South Asia.
Gergen [8]	An Invitation to Social Construction	Argues that cultural narratives shape help-seeking behaviors, reinforcing the preference for traditional healing in Nagaland.	Theoretical contribution; applied here as a framework. It helps situate Naga practices within constructivism.
Gill, & Singh [9]	Tribal Mental Health and Belief Systems in India	The article examines the mental health beliefs and access to care among India’s tribal communities. The review, published in June 2023, analyzes studies from databases like JSTOR, Google Scholar, NCBI, PubMed, and Scopus, focusing on research published between 2016 and 2022. It highlights the scarcity of community-based research on mental health concerns within these populations	Recent and highly relevant, but still broad Nagaland is part of a larger analysis rather than the exclusive focus.
Hansda, Singh, Kapse, & Kiran [11]	Supernatural Attitude and Mental Health Practices among the Tribal with Special Reference to Jharkhand.	The study provides valuable insights into the mental health beliefs and practices of tribal communities in Jharkhand, India. The research highlights that these communities often attribute mental illness to supernatural causes such as ghosts, black magic, or divine curses. It also identifies psychosocial issues like alcoholism, unemployment, debt, family disputes, inferiority complex, poverty, illiteracy, and malnutrition as contributing factors to mental health problems. The study emphasizes the need for community-based awareness programs to educate tribal populations about mental health, suggesting that such initiatives be conducted in collaboration with health workers and local communities, particularly in weekly marketplaces.	Provides comparative context to Nagaland, but not region-specific. Helps strengthen the case for shared tribal frameworks.

### Implications for culturally sensitive mental health interventions

The findings of this review emphasize the importance of incorporating cultural perspectives into mental health interventions. Although this review takes a social constructivist position, we are aware of the relativist limitation in this approach that cross-cultural comparison is problematic. To overcome this, we incorporate aspects of critical realism, which recognizes both the socially constructed nature of meaning and the reality of underlying structures of human experience. Likewise, existential approaches (Yalom [25]; Vontress & Epp, 2015) propose that some universal givens mortality, isolation, meaning, for example; give a shared structure across cultures. This two-way lens allows comparison of indigenous idioms of distress and Eurocentric psychiatric categoriesThe integration of traditional beliefs and practices into therapeutic approaches can help bridge the gap between indigenous healing systems and modern psychiatric care.

1. **Cultural Sensitivity in Mental Health Practices** .
2. For mental health professionals working in Nagaland, it is essential to adopt culturally sensitive approaches that respect the beliefs and practices of the local communities. This may involve collaborating with traditional healers and incorporating indigenous healing practices alongside modern treatment methods [8]. Mental health practitioners should recognize lycanthropy as both a locally meaningful idiom of distress and a manifestation of broader human concerns. Integrating indigenous healing practices with evidence-based psychiatric care can reduce stigma and enhance treatment relevance. A culturally hybrid approach allows practitioners to validate spiritual interpretations while also providing biomedical explanations where appropriate.
3. **Policy Implications** .
4. Policymakers should consider the cultural context when designing mental health programs and services in Nagaland. This includes training mental health professionals in cultural competence and promoting the integration of traditional and modern approaches to care (Kirmayer & Bhugra, 2021). Furthermore, community-based mental health interventions that engage local leaders and healers may help reduce stigma and encourage help-seeking behavior.

This study emphasizes the use of social constructivism as a framework for understanding the cultural constructs of distress and lycanthropy in Nagaland. Although this review takes a social constructivist position, we are aware of the possible relativist flaw in this position whereby cross-cultural comparison is at risk. To counteract this, we also utilize aspects of critical realism (Bhaskar, 2016), which embraces both the socially constructed character of meaning and the presence of underlying structures of human experience. In the same vein, existential views (Yalom [25]; Vontress & Epp, 2015) propose that there are some universal givens like mortality, solitude, and meaning that present a shared template within cultural contexts. This two-pronged approach allows indigenous idioms of distress to be compared against Eurocentric psychiatric constructs.

Social constructivism, as articulated by Berger and Luckmann [4], argues that reality is constructed through social interactions and shared meanings within a particular cultural context. In Nagaland, this lens reveals how cultural beliefs, local healing practices, communal narratives, and spiritual frameworks collectively shape the understanding of mental health and distress, particularly phenomena like lycanthropy.

## Social constructivism and cultural constructs of distress

The social constructivist theory highlights how distress is not universally defined but is instead shaped by the social, cultural, and spiritual contexts within a given community. In Nagaland, distress is often seen as stemming from spiritual disturbances, ancestral discontent, or supernatural causes, and this view is passed down through generations. As Kirmayer & Bhugra [15] point out, psychological suffering is understood in relation to the local belief systems, traditions, and narratives. The constructivist approach emphasizes that the meanings ascribed to distress in Nagaland are deeply rooted in its historical and social context, rather than being inherent to the individual’s psychological state.

## Role of indigenous healing practices in the social construction of distress

Social constructivism also emphasizes the significance of local healing practices in defining and addressing distress. Indigenous healers in Nagaland do not merely treat symptoms of mental distress but rather intervene in the social construction of distress itself. As discussed, these healing practices often involve spiritual rituals, offerings, and communing with ancestors or spirits, which are viewed as essential components of the healing process. From a constructivist perspective, these rituals help to reframe and recontextualize distress, positioning it within a framework that makes sense to the community and the individual suffering from it. This cultural redefinition of distress is crucial for understanding the effectiveness of local healing practices.

## Lycanthropy and social constructivist perspectives

Lycanthropy, typically seen in Western psychiatric frameworks as a delusional disorder, takes on a different significance in Nagaland. Through a social constructivist lens, lycanthropy can be understood not as a mental illness but as a cultural expression of distress, influenced by the societal narratives and spiritual beliefs prevalent in the region.Khesheli Aye’s research, titled “Human-Animal Transitions: A Case Study of Sümi Lycanthropy”, explores the belief in human-animal transformations among the Sümi Naga tribe. Aye discusses how these beliefs are deeply embedded in the cultural and social frameworks of the community, functioning as a shared system that provides meaning to individuals’ experiences.​.

Similarly, Rebekka Sutter’s work, “Tigermen in Nagaland: Collective Imagination or Intimate Knowledge of Other Worlds”, examines the phenomenon of tiger transformation among Naga communities. Sutter highlights that such beliefs are not merely individual experiences but are collectively upheld within the community, emphasizing lycanthropy’s role as a culturally constructed phenomenon.

## Interplay between modern psychological frameworks and social constructs

Longkumer (2020) explores the tension between modern psychiatric frameworks and indigenous social constructs in understanding mental health. While modern psychology may pathologize lycanthropy, the constructivist approach emphasizes the importance of recognizing local perspectives. The social constructivist framework advocates for integrating indigenous healing practices with contemporary psychological approaches, acknowledging that the intersection of modern and traditional belief systems provides a fuller understanding of distress and mental illness in Nagaland.

## Social constructivism in mental health

Using social constructivism to explore mental health in Nagaland allows us to understand how mental health concepts are not static or universal, but rather, are constructed through social processes. Berger & Luckmann [4] argue that the realities we perceive are shaped by social interactions and shared experiences. In Nagaland, these shared experiences include the community’s understanding of distress, the societal responses to it, and the healing processes that arise from these beliefs. Social support systems, including family, religious leaders, and community healers, are integral to the cultural construction of distress and the coping mechanisms employed.

Conclusion: Social constructivist insights into distress and lycanthropy..

In conclusion, this study emphasizes the importance of social constructivism as a critical framework for understanding the cultural constructs of distress in Nagaland, particularly the phenomenon of lycanthropy. The research shows that distress is a socially constructed experience, deeply embedded in the spiritual beliefs, cultural narratives, and local healing practices of the region. By acknowledging that the meaning of mental health is context-dependent, this approach helps bridge the gap between Western psychiatric models and indigenous perspectives on distress.

The integration of indigenous healing practices with modern psychological frameworks offers a more holistic and culturally sensitive approach to mental health. It also highlights the importance of understanding the social context in which mental health issues, like lycanthropy, are constructed, perceived, and addressed (Table 4).

**Fig 2:**
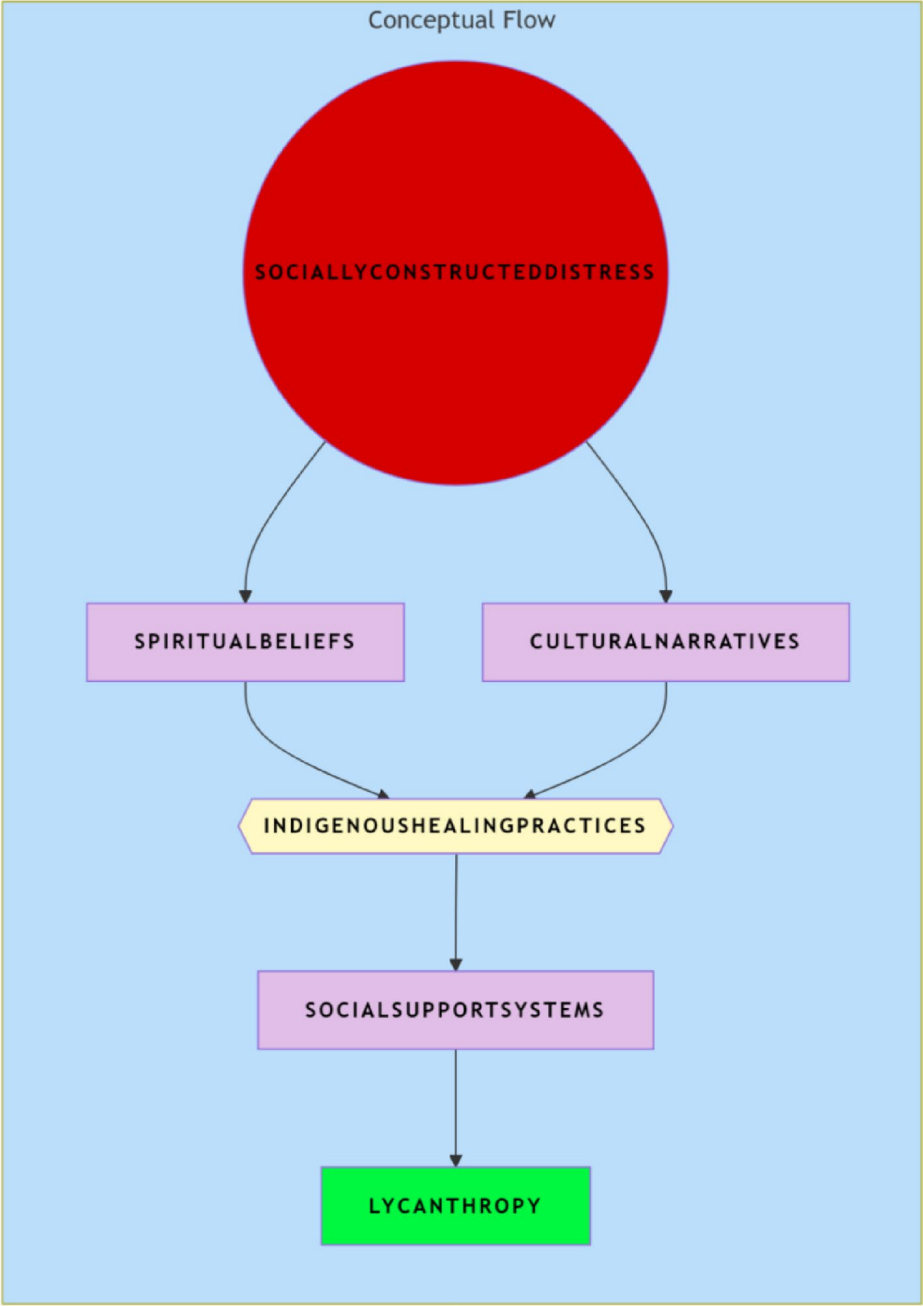
Conceptual Framework of Socially Constructed Distress and Lycanthropy in Nagaland

Thus, social constructivism provides a valuable lens for interpreting distress not as a purely individual phenomenon but as a culturally mediated experience, shaped by social interactions and shared beliefs within the community (Fig. 2)

- Socially Constructed Distress.
- Indigenous Healing Practices.
- Lycanthropy.

**Table 4:** Supporting literature

Author & year	Title	Key findings	Critical notes
Laskar [18]	Indigenous Knowledge and Traditional Use of Medicinal Plants by Four Major Tribes of Nagaland, North East India	Indigenous healing practices in Nagaland involve the use of 257 species of medicinal plants by four major tribes: Ao, Angami, Lotha, and Sema. Local healers utilize various plant parts, primarily leaves, roots, and fruits, to prepare remedies through methods like decoction and paste. These practices are deeply rooted in tradition, with healers treating multiple ailments using specific plants. However, there is a concern about the erosion of this knowledge among younger generations, necessitating conservation efforts and community involvement.	Strong empirical detail on indigenous healing resources; however, limited connection to psychological constructs of distress.
[22]	Conservation of folk healing practices and commercial medicinal plants with special reference to Nagaland	This paper explores the conservation of traditional healing practices among the tribal people of Nagaland, a biodiversity-rich state in North Eastern India. The study highlights how herbalists use single or compound formulations for treatment. However, modernization is causing a decline in these practices. The authors emphasize the need for conservation by integrating traditional healers into scientific research and validating their methods through scientific organizations.	Valuable for linking biodiversity with healing, but largely ecological/medical rather than psychological in focus.
[7]	Cosmology, Ecology, and Spirituality: Reappraising Stone Culture, Genna Tradition, and Ancestor Veneration among the Naga Tribes	This article reappraises Naga cosmology and spirituality, which are deeply embedded in ecology, folklore, stone culture, gennas (rituals and taboos), and ancestor veneration. It highlights the enduring spiritual connection between the Naga people, their land, and the supreme creator. The study also emphasizes the roles of shaman-priests, clan elders, and agricultural rituals in maintaining village customs and ecological harmony.	Provides important cultural depth; limitation lies in its thematic rather than clinical analysis.
Gergen [8]	An Invitation to Social Construction	Explores the social constructivist theory, which supports the understanding of lycanthropy as a culturally specific phenomenon.	Strong theoretical foundation, but abstract. Requires contextualization to Nagaland’s cultural psychiatry.
[17]	Cultural concepts of distress and psychiatric disorders: literature review and research recommendations for global mental health epidemiology	This study reviews the relationship between Cultural Concepts of Distress (CCD) and psychiatric categories, assessing 45 studies with 18,782 participants. Using the **SAQOR-CPE** framework, it finds most studies are of low quality, with methodological flaws affecting results. Meta-analyses show high variability, with stronger CCD-psychiatric disorder links in weaker studies. The authors argue that **better study design** can enhance mental health diagnostics, reduce cultural bias, and improve intervention relevance.	Widely cited methodological benchmark; however, global in scope, with limited reference to Northeast India.
Amineh, & Davatgari [2]	Review of Constructivism and Social Constructivism	Amineh and Davatgari Asl [2] emphasize that culture plays a crucial role in learning, especially in social constructivism. They argue that knowledge is shaped by social interactions within a learner’s cultural environment, influencing how information is interpreted and internalized. In education, especially EFL teaching, understanding cultural backgrounds helps create effective learning strategies by making content more meaningful and engaging for students.	Useful educational perspective, though not directly tied to mental health. Applied here as theoretical support.
Agius [1]	Social constructivism	Christine Agius, in Chap. 6 of *Contemporary Security Studies*, explores social constructivism’s role in shaping security concepts. She argues that security threats are not objective but are socially constructed through ideas, identities, and interactions. Drawing on Wendt’s notion that “anarchy is what states make of it,” she highlights how state behavior is influenced by shared understandings rather than fixed realities. Culture plays a crucial role in this process by shaping national identities, norms, and perceptions of security threats. Different cultural contexts lead to varied security priorities and interpretations of global issues, reinforcing the idea that security is constructed rather than universally defined.	Cross-disciplinary illustration of constructivism; useful for conceptual framing but indirect relevance to mental health.

## Conclusion

In this study, a social constructivist analysis of cultural constructs of distress and lycanthropy in Nagaland reveals how communal belief systems, spiritual frameworks, and localized social norms shape mental health perceptions. Rather than viewing lycanthropy merely as a delusional or pathological phenomenon, this perspective highlights its cultural and spiritual significance within Naga society. Distress in Nagaland often emerges from a fusion of ancestral beliefs, supernatural interpretations, and community-based coping strategies realities that do not always align with Western psychiatric models.

By examining these narratives through the lens of social constructivism, the research emphasizes the importance of understanding mental health in its specific cultural context. Indigenous healing practices, which frequently involve ritual and spiritual intervention, remain deeply relevant despite the growing influence of modern psychiatric approaches. The coexistence of these two systems indigenous and biomedical can create tensions, yet it also offers avenues for more holistic mental health interventions.

Ultimately, this study suggests that effective mental health care in Nagaland must integrate local spiritual and communal dimensions alongside contemporary psychological frameworks. Embracing cultural narratives and shared belief systems not only respects the lived experiences of individuals but also enhances the acceptability and impact of interventions. Moving forward, collaborations between traditional healers, community leaders, and mental health professionals will be crucial in bridging indigenous practices and modern clinical methods. Such culturally sensitive approaches can help reduce stigma, improve help-seeking behaviors, and foster a richer, more nuanced understanding of distress and lycanthropy in the region .

## Data Availability

No datasets were generated or analysed during the current study.
